# Simultaneous Screening and Validation of Effective Zinc Finger Nucleases in Yeast

**DOI:** 10.1371/journal.pone.0064687

**Published:** 2013-05-31

**Authors:** Ling Wang, Juan Lin, Tingting Zhang, Kun Xu, Chonghua Ren, Zhiying Zhang

**Affiliations:** College of Animal Science and Technology, Northwest A & F University, Yangling, Shaanxi, China; Center for Genomic Regulation, Spain

## Abstract

Zinc finger nucleases (ZFNs) have been successfully used for genome modification in various cell types and species. However, construction of an effective ZFN remained challenging. Previous studies all focused on obtaining specific zinc finger proteins (ZFPs) first via bacterial 2-hybrid approach, and then fusing selected ZFPs to FokI nuclease domain. These assembled ZFNs have high rate of failing to cleave target sites *in vivo*. In this study, we developed a simultaneous screening and validation system to obtain effective ZFNs directly in yeast AH109. This system is based on Gal4 reporter system carrying a unique intermediate reporter plasmid with two 30-bp *Gal4* homology arms and a ZFN target site. DNA double strand breaks introduced on target sequence by ZFNs were repaired by single strand annealing (SSA) mechanism, and the restored Gal4 drove reporter genes expression. Taking the advantage of OPEN (Oligomerized Pool ENgineering) selection, we constructed 3 randomized ZFNs libraries and 9 reporter strains for each target gene. We tested this system by taking goat *α s1-casein* as target gene following three-step selection. Consequently, 3 efficient pairs of ZFNs were obtained from positive colonies on selective medium. The ZFNs achieved a 15.9% disruption frequency in goat mammary epithelial cells. In conclusion, we created a novel system to obtain effective ZFNs directly with simultaneous screening and validation.

## Introduction

Zinc finger nucleases (ZFNs) as artificial enzymes exhibit extraordinary success in genome engineering. These enzymes are composed of a designed polymeric zinc finger protein (ZFP) and the nuclease domain of FokI restriction endonuclease [1], [2]. ZFPs provide the ability to recognize and bind particular DNA sequences with two FokI domains as a dimmer generating DNA double strand breaks (DSBs) [3]. ZFN-introduced DSBs can abrogate gene functions through imprecise repair of non-homologous end joining (NHEJ) or introduce customized change by homologous recombination (HR) repair from a supplied donor DNA [4].

ZFNs-mediated genomic modification has been successfully demonstrated in a variety of plants and animals, including maize [5], *C.elegans* [6], *Drosophila* [7], *Xenopus* [8], zebrafish [9], [10], mouse [11], [12], rat [13] as well as human ES (embryonic stem) and iPS (induced pluripotent stem) cells [14]. ZFNs have also shown potential in gene therapy: ZFN-modified human CD4^+^ T cells with permanent resistance to HIV infection are currently in phase 2 clinical trials for treatment of AIDS [15].

The target specificity of ZFNs is largely determined by ZFPs, which contain a tandem array of multiple fingers [16]. Each zinc finger motif primarily recognizes a 3-bp DNA sequence. For classical Cys2His2 zinc finger motif, four key amino acids at position −1, 2, 3, 6 relative to the start of the α-helix contribute most to the interaction specificity [17]. By changing key residues of zinc fingers, ZFN binding specificity can be altered providing ZFPs with different sequence specificities [18].

The wide-use of ZFNs is mainly limited by the challenge of creating ZFNs with publicly available sequences that confer the specificity for a desired genomic target region. Three major platforms for generation of ZFPs with customized specificity exist: (1) Modular assembly (MA) [19], (2) Oligomerized Pool ENgineering (OPEN) [20], (3) Context-dependent assembly (CoDA) [21]. The three approaches were originally developed by Zinc Finger Consortium and are open to the public. MA is an easy approach to obtain ZFPs. However, it ignores the context-dependent interactions between fingers. Consequently, ZFNs obtained by MA show a high failure rate of 73.1% [23] which can be optimized via the two-finger archive [22]. OPEN system combines large scale pre-selection ZFP libraries with the two-round selection via bacterial two-hybrid. Although OPEN is laborious and time-consuming, OPEN ZFNs demonstrated high efficiency and specificity [24]. CoDA is based on the performance of zinc finger motifs of known specificity, whereby 3-finger arrays are assembled with mediation of the zinc finger 2. CoDA-generated ZFNs demonstrate a favorable ability to cleave target sites in zebrafish and plants with a success rate of 50% [21], but the limited number of defined ZFPs precludes its wide deployment in complex genomes.

The three platforms described above have a similarity of obtaining ZFPs first and subsequently incorporating them into ZFNs. As a consequence, some ZFNs fail to target gene of interest *in vivo*, even if ZFPs have high DNA binding affinity *in vitro* [23]. This necessitated further efforts to generate functional ZFNs. Two reports developed yeast-based assays to test obtained ZFNs activities through detectable reporter genes *LacZ* or *MEL1* [9], [25], and illustrated that yeast was a suitable host to generate effective ZFNs.

Here, we demonstrate a comprehensive approach of simultaneous screening and validating specific ZFNs in yeast. Briefly, based on Gal4 reporter system and OPEN method, efficient ZFNs were screened from randomized libraries and activity of ZFNs were assessed during the process of screening. ZFNs generated by this system were used in target cells directly and demonstrated robust DNA cleavage ability. Collectively, we present here a platform for a more rapid and simultaneous screening and validation of ZFNs in yeast that opens attractive avenues to achieving efficient customized nucleases.

## Materials and Methods

### Yeast Strain and Medium

The yeast *Saccharomyces cerevisiae* strain used in this study was AH109 (MATa, trp1-901, leu2-3, 112, ura3-52, his3-200, gal4Δ, gal80Δ,LYS2 : : GAL1_UAS_-GAL1_TATA_-HIS3, GAL2_UAS_-GAL2_TATA_-ADE2, URA3 : : MEL1_UAS_-MEL1_TATA_-LacZ MEL1) (Clontech), in which Gal4-responsive upstream activating sequences (UASs) and TATA boxes control four integrated reporter genes, *HIS3,ADE2,Mel1* and *LacZ*. Yeast rich medium YPD contained 1% yeast extract, 2% peptone, 2% glucose, and 1.5% bacto agar when preparing for plates. Selective medium contained 0.67% yeast nitrogen base, 0.06% complete dropout amino acid mixture (lacking leucine, tryptophan, adenine, and histidine), 2% glucose, 50 µg/ml G418 and 1.5% bacto agar. Non-selective medium was SD medium adding histidine, adenine, or tryptophan if necessary.

### Yeast Transformation and Plasmid Extraction

High efficiency yeast transformation was performed by the lithium acetate method as Gietz *et al* described [26]. Plasmids in yeast cells were extracted via yeast plasmid extraction kit by standard procedure (Omega Bio-tek, Georgia, USA). ZFN encoding plasmids from pLeu- FokI were isolated on LB plates with 100 µg/mL Ampicillin, and plasmids from pTrp- FokI were isolated on LB plates with 50 µg/mL kanamycin.

### Cell Culture and Electroporation

Goat mammary epithelial (GME) cells [27] were grown in D/F-12 (Hyclone) with 5 mg/L insulin (Sigma), 1 mg/L hydrocortisone (Sigma), 10% fetal calf serum (Hyclone) and 1% penicillin/streptomycin, and maintained at 37°C and 5% CO_2_.

Plasmid DNA was delivered into GME cells by electroporation with the device of ECM 2001 (BTX). One million cells were kept in 400 µl of electroporation buffer [28], with optimized condition of 220 volts, 1 ms, 3 times. Unless indicated otherwise, the amount of DNA used for electroporation was 10 µg of each ZFN or empty control expression vector and 20 µg of reporter vector.

### Construction of Reporter Plasmids and Selection Strains

**Figure 1:**
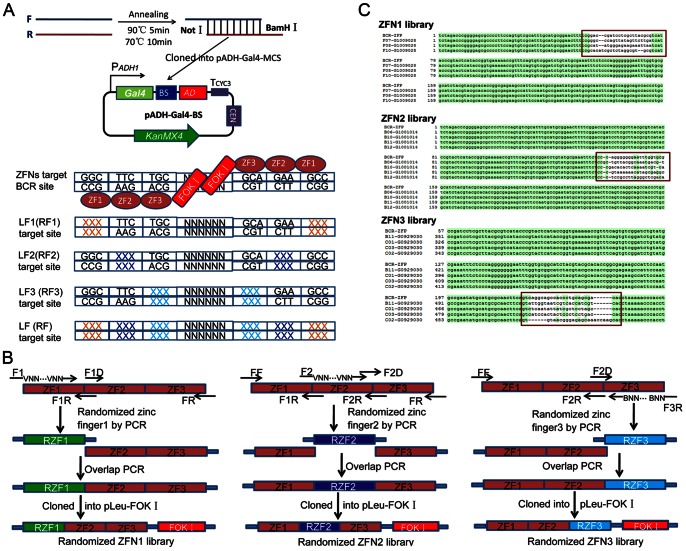
Construction of reporter vectors and randomized ZFN libraries. (**A**) **Schematic of constructing reporter plasmids.** Target sites with *Not*I/*Bam*HI sticky ends were generated via two oligonucleotides direct annealing (90°C 5 min, 70°C 10 min, cool down to room temperature), and inserted into plasmid pADH-Gal-MCS, resulting in reporter vector pADH-Gal4-BS. Reporter plasmids containing palindromic sequences of target sites for screening three 3-bp subsites of left (right) were substituted by BCR subsites, respectively. 9-bp half sites took place of BCR target sequence completely in LF (left half) and RF (right half) target sites. (**B**) **Illustration of constructing 3 randomized ZFN libraries.** The randomized finger 1 fragments were amplified with primers F1/F1R, finger 2 and finger 3 were amplified with primers F1D/FR and all 3 fingers were fused together by overlap PCR. PCR products were cloned in yeast expression plasmids pLeu-FokI between *Xba*I/*Bam*HI sites resulting in randomized ZFN1 library. The construction of randomized ZFN2 and ZFN3 libraries was similar to that of ZFN1 library. (**C**) **Sequence alignment results of randomized ZFN libraries.** The regions of red cube represent sequences of randomized zinc fingers in each library in which DNA sequences of key amino acids were mutated by PCR with randomized primers and the other two zinc fingers were fixed to the corresponding fingers of BCR-ZFP.

Potential target sites in gene of interest were identified by web-based ZiFiT software [29]. According to OPEN selection, one 3-bp subsite of three was substituted for a corresponding BCR subsite, and the other two subsites were fixed [24]. Mixture of two complementary oligonucleotides carrying target sites was incubated in 90°C for 5 min, 70°C for 10 min and cooled down to room temperature. The annealing products with *Not*I/*Bam*HI sticky ends were cloned into pADH-Gal4-MCS reporter vector (Figure 1A). The characteristics of OPEN selection demonstrated that separate 3 reporter vectors for a 9-bp half target site were used in the first step screening. For the second step screening, two reporter vectors harboring two palindromic sequences of 9-bp left (right) half target sites were utilized. The reporter harboring 24-bp full length target site was used for the third step screening. The primers used for constructing reporter plasmids are listed in Table 1. After sequencing confirmation, 0.5 µg reporter plasmids were transformed into AH109, and surviving colonies on YPD plates with G418 were isolated and used as selection strains for screening.

**Table 1:** Primers used for construction of reporter plasmids.

Primer Name	Primer sequence (5′-3′)
LF1BSF	GGCCGCAACTTCTGCTGATAGCAGAAGTTG
LF1BSR	GATCCAACTTCTGCTATCAGCAGAAGTTGC
RF1BSF	GGCCGCGTTTTCTGCTGATAGCAGAAAACG
RF1BSR	GATCCGTTTTCTGCTATCAGCAGAAAACGC
LF2BSF	GGCCGCGGCAGCTGCTGATAGCAGCTGCCG
LF2BSR	GATCCGGCAGCTGCTATCAGCAGCTGCCGC
RF2BSF	GGCCGCGGCCACTGCTGATAGCAGTGGCCG
RF2BSR	GATCCGGCCACTGCTATCAGCAGTGGCCGC
LF3BSF	GGCCGCGGCTTCCTATGATATAGGAAGCCG
LF3BSR	GATCCGGCTTCCTATATCATAGGAAGCCGC
RF3BSF	GGCCGCGGCTTCTGCTGATAGCAGAAGCCG
RF3BSR	GATCCGGCTTCTGCTATCAGCAGAAGCCGC
LBSF	GGCCGCAACAGCCTATGATATAGGCTGTTG
LBSR	GATCCAACAGCCTATATCATAGGCTGTTGC
RBSF	GGCCGCGTTCACTGCTGATAGCAGTGAACG
RBSR	GATCCGTTCACTGCTATCAGCAGTGAACGC
BSF	GGCCGCAAACAGCCTATGATAGCAGTGAACCG
BSR	GATCCGGTTCACTGCTATCATAGGCTGTTTGC

### Construction of 3 Randomized Zinc Finger Nucleases Libraries

Three independent libraries were constructed from a standard frame of BCR-ZFP [30], in which three zinc fingers have the ability to target gene encoding breakpoint cluster region (BCR) protein. In each library, key amino acids at position −1 to +6 relative to the start of the α-helix in one finger were altered by 7 degenerate codons (5′-VNN-3′), and the other two fingers were kept constant to BCR-ZFP. The mutated zinc fingers were amplified with randomized primers containing 7 VNN codons (Table 2), and the other two BCR zinc fingers were amplified with universal primers. Then, zinc finger segments were assembled by overlap PCR to generate 3-zinc finger arrays. Subsequently, PCR products were ligated into the ZFN expression vector pLeu- FokI between *Xba*I/*Bam*HI sites, such that FokI domain was fused at the C-terminus of the three zinc fingers. After electroporation into DH5α, 1×10^7^∼1×10^8^ cells were pooled, resulting in randomized ZFN1, ZFN2 and ZFN3 libraries (Figure 1B). Primers used in construction of randomized ZFNs libraries are listed in Table 2.

**Table 2:** Primers used for construction of randomized ZFNs libraries.

Primer Name	Primer sequence (5′-3′)	Description
F1	TGCATGCGGAACTTTTCGVNNVNNVNNVNNVNNVNNVNNCATACCCGTACTCATACC	Forward primer for randomized ZF1
F2	TGTATGCGAAATTTCTCCVNNVNNVNNVNNVNNVNNVNNCATCTACGTACGCACACC	Forward primer for randomized ZF2
F3R	AGGTGGGTTTTTAGGTGNNBNNBNNBNNBNNBNNBNNBACTGAAGTTGCGCATGCA	Forward primer for randomized ZF3
F1D	CATACCCGTACTCATACC	Forward primers for BCR-ZF2
F1R	GGAGAAATTTCGCATACA	Reverse primers for BCR-ZF1
F2D	CATCTACGTACGCACACC	Forward primers for BCR-ZF3
F2R	ACTGAAGTTGCGCATGCA	Reverse primers for BCR-ZF2
FF	GGCTCTAGACCCGGGGAGCGCCCCTTCCAGTGTCGCATTTGCATGCGGAACTTTTCG	Forward primer with *Xba*I
FR	GCCGGATCCCCTCAGGTGGG	Reversed primer with *Bam*HI

### Screening for Specific ZFNs

**Figure 2:**
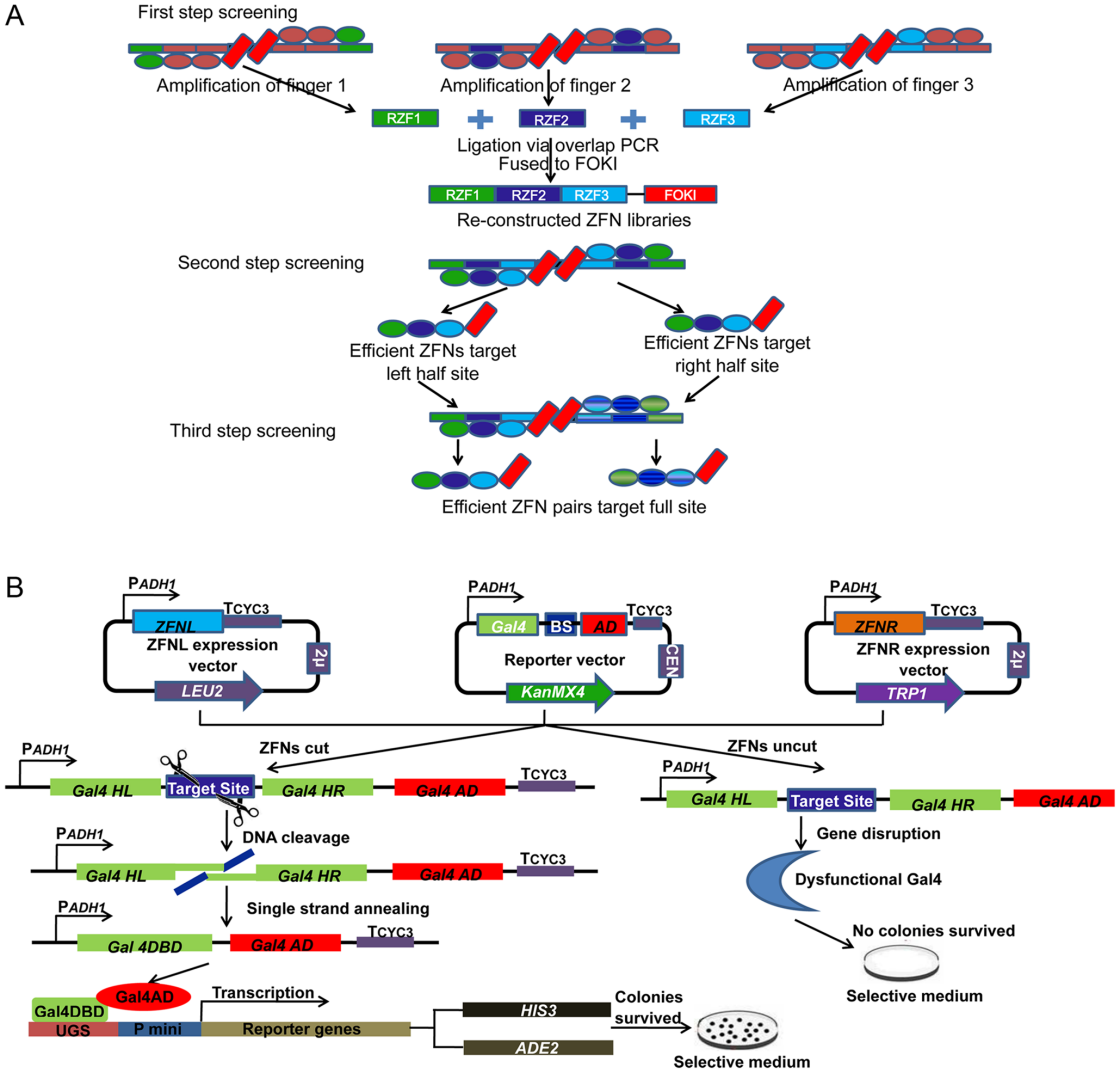
Schematic diagram of yeast-based ZFNs screening and validation system. (**A**) **Schematic representation of simultaneous screening and validation of ZFNs in yeast.** Host strain AH109 harbored ZFNs expression and reporter plasmids and co-expressing ZFNs that could cut target sites on *Gal4* to generate double strand breaks (DSBs). DSBs were repaired via cellular single strand annealing (SSA) and *Gal4* was restored by removing the target site and one homology region. Thus, functional Gal4 started to drive expression of reporter genes in AH109, and yeast colonies survived on selective medium. By comparison, ZFNs had no abilities to cut target sites and dysfunctional *Gal4* could not drive reporter genes expression either. Therefore, yeast colonies could not survive on selective medium lacking histidine and adenine. Gal4HL: Gal4 homology left arm, Gal4HR: Gal4 homology right arm, Gal4AD: Gal4 active domain, Gal4DBD: Gal4 DNA binding domain. (**B**) **The procedure of screening efficient ZFNs via three-step selection.** The first step aimed at enriching efficient ZFNs target 3-bp subsites from randomized ZFNs libraries. 3 enriched single fingers target 9-bp half sites were amplified and assembled by overlap PCR and cloned into vector pLeu-FokI between *Xba*I/*Bam*HI sites to generate re-constructed ZFNs libraries. The second step screening aimed at screening for efficient ZFNs target 9-bp half sites from re-constructed ZFNs libraries. Finally, pairs of efficient ZFNs were obtained after the third step screening. RZF1: Enriched randomized-zinc finger 1, RZF2: Enriched randomized-zinc finger 2, RZF3: Enriched randomized-zinc finger 3.

In this system, 3-step selection was deployed to obtain effective ZFNs for target sites of interest. The selection of single finger binding to each 3-bp subsite was performed in the first step screening. And 1 µg plasmids encoding ZFN libraries were transformed into corresponding selection strains. Transformants were plated on non-selective and selective SD plates, and maintained in 30°C for 3 days (Figure 2B). Subsequently, ZFN expression vectors in surviving colonies were recovered from selective plates. For left or right half sites, 3 enriched ZFN libraries targeting each 3-bp subsite were obtained. Three zinc fingers were amplified from enriched ZFN encoding plasmids with universal primers (Table 2), and assembled by overlap-PCR to generate re-constructed ZFN left and right libraries.

In the second step screening, 1 µg plasmids of re-constructed ZFN left and right libraries were transformed into selection strains LBS and RBS, respectively. Plasmids encoding ZFNHL target left half site and plasmids encoding ZFNHR target right half site were recovered from surviving colonies on selective SD plates as candidate ZFNs for further selection (Figure 2B).

Finally, 1 µg both ZFNHL and ZFNHR expression plasmids were co-transformed into selection strain FBS bearing the 24-bp full target site. The surviving colonies contained candidate ZFN pairs targeting the designed 24-bp site (Figure 2B). Pairs of ZFN expression vectors in yeast were recovered and amplified in *E.coli* DH5α.

### Detection of NHEJ-mediated Mutations in GME Cells

For further determination of ZFNs cleavage activity in target cells, we cloned ZFN pairs into modified pST1374-sharkey [31] with *Not*I/*Bam*HI sites to generate ZFNs mammalian expression vectors. A reporter plasmid carried a *puromycin resistance* (*PuroR*) gene expression cassette in which two divided *PuroR* segments were separated by a ZFN target site. Both *PuroR* segments contained a 300-bp homology region. Upon cleavage at the ZFN target site, the cellular single strand annealing (SSA) repair mechanisms remove one homology arm [33] and the target site and restore the wild-type *PuroR* expression cassette. Therefore, PuroR expression confers a resistance to puromycin antibiotics and serves as surrogate for enrichment of genome-modified positive cells.

ZFNs expression vectors and reporter plasmids were co-transfected into GME cells by electroporation. 5 µg/mL puromycin (Sigma) was added into culture 2 days post electroporation and genomic DNA was extracted from the surviving clones post 5 days of puromycin treatment. Primers CAF (5′-AAAAGCTATGCCAATTTCAATCA-3′) and CAR (5′-GATTCCTATCAAAAGCTATGCCA-3′) were used to amplify a 400-bp fragment surrounding the target site from genomic DNA. PCR products were purified and then denatured and re-annealed via the following program: 95°C for 10 min; 95°C to 85°C cooling at a rate of −2°C/sec; 85°C to 25°C cooling at a rate of −0.1°C/sec; rapid cool to 4°C. Hybrid duplexes were treated with T7 endonuclease I (T7 E I, NEB) assay and then detected by electrophoresis on 3% agarose gel. Endogenous locus disruption percentage was determined as described previously [32]. PCR fragments were cloned into pGEM-T for sequencing to illustrate ZFN-induced indels.

## Results

### Simultaneous Screening and Validation of an Efficient ZFNs System

Our yeast screening and validation system contains a unique reporter vector bearing a designed target site and plasmids encoding ZFN pairs. The reporter vector has three critical elements (Figure 1A): (1) target site located between two 30-bp homology arms, (2) *KanMX4* expression cassette for G418 selection, (3) yeast-derived replicated centromere (CEN) element for a low copy number in yeast, which makes the copy of target site is similar with yeast genome. ZFN-mediated DNA double strand breaks (DSBs) at the target sites have been repaired precisely via cellular SSA mechanism in the presence of two *Gal4* homology arms [33]. Subsequently, functional Gal4 was restored and drove selectable markers (yeast *HIS3* and *ADE2* genes) expression. Positive colonies could survive on histidine- and adenine-deficient selective medium. By contrast, ZFNs without the ability to target the designed sites could not generate DSBs on reporter vectors. As a consequence, dysfunctional Gal4 could not activate the reporter genes transcription and no colonies survived on selective medium (Figure 2A). This system provides an alternative method to screen effective ZFNs as well as validate activities of assembled ZFNs.

### Three Randomized ZFNs Libraries

Seven key amino acids in a finger were randomized using degenerate triplets (5′-VNN-3′), and the other two fingers were constant to BCR-ZFP (Figure 1B). According to limited dilution, the complexity of ZFN1 library was ∼1.2×10^8^ members, the complexity of ZFN2 library was ∼3.0×10^8^members and the complexity of ZFN3 library was ∼2.3×10^8^ members. DNA sequence alignment was performed among ZFP sequences of libraries and BCR-ZFP, and results demonstrated that sequences of randomized fingers were totally different from BCR-ZFP and mismatches also existed among fingers in the same library, while the other two fingers were fixed to BCR-ZFP (Figure 1C). This indicates that recognition helix residues –1 to 6 of zinc fingers in libraries were altered by randomized primers, as expected.

### Screening for Efficient ZFNs Target 3-bp Subsites

**Figure 3:**
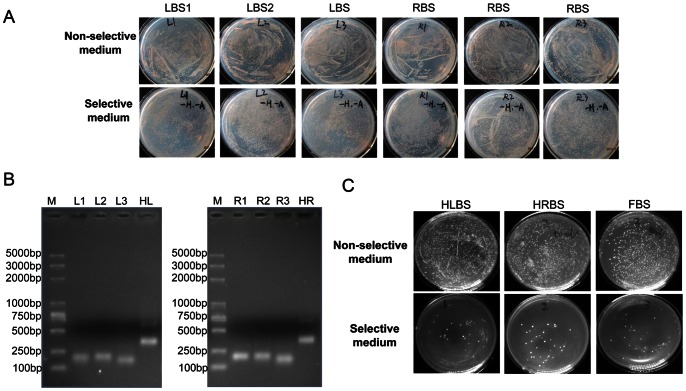
Screening efficient ZFNs target goat alpha s1-casein gene via this yeast-based system. (**A**) **The first step screening for six 3-bp subsites.** Plasmids encoding randomized ZFN libraries were transformed into corresponding selection strains. And 10 µl transformants were plated on non-selective medium to test transformation efficiency and 100 µl transformants were plated on selective medium to obtain efficient ZFNs. LBS1(RBS1), LBS2(RBS2) and LBS3(RBS3) selection strains were transformed with appropriately randomized ZFN1, ZFN2 and ZFN3 libraries, respectively, to enrich fingers binding to three left (right) 3-bp subsites. (**B**) **Amplification of enriched 3 zinc fingers and assembly via overlap PCR.** Surviving colonies were scraped from selective plates, and plasmids encoding ZFNs were recovered from yeast. PCR was performed to amplify enriched fingers from enriched ZFNs plasmids. Left picture represents amplification of enriched 3 zinc fingers with 3-finger arrays targeting left half target site. Right picture represents amplification of enriched 3 fingers with 3-finger arrays targeting right half target site. Three individual fingers were assembled by overlap PCR with primers FF/FR. (**C**) **Screening for ZFNs target two half sites and the full length site.** LBS and RBS selection strains harboring left half site (5′-TAG GCT GTT-3′) and right half site (5′-GCA GTG AAC-3′) were transformed with re-constructed left and right ZFNs libraries, respectively. Plasmids encoding efficient ZFNHL and ZFNHR were recovered from survival yeast colonies on selective plates. FBS selection strain harboring full target site (5′-AAC AGC CTA TGATA GCA GTG AAC-3′) was co-transformed with ZFNHL and ZFNHR expression vectors. Plasmids encoding efficient ZFN pairs were recovered from yeast colonies on selective plates.

Nine reporter strains harboring goat *α s1-casein* target sites were obtained as previously described (Figure 1A). In the first step screening, 1 µg plasmids encoding randomized ZFN libraries were transformed into their corresponding selection strains (Figure 2). Ten microliters of transformants were plated on non-selective medium to test transformation efficiency and 100 µl of transformants were grown on selective plates. Three days post transformation, about 10% of transformants survived on selective medium (Figure 3A). Plasmids encoding enriched ZFNs were recovered from colonies on selective SD plates. Three fingers RZF1, RZF2 and RZF3 were amplified from plasmids encoding enriched ZFNs target three 3-bp subsite, and joined by overlap PCR (Figure 3B). Then PCR products were cloned into plasmid pLeu-FokI between *Xba*I/*Bam*HI sites to generate re-constructed ZFN libraries for left and right half sites.

### Screening ZFNs Target Half and Full Sites

The selection for efficient ZFNHL (ZFNs target left half site) and ZFNHR (ZFNs target right half site) target 9-bp left and right half sites was performed in the second step screening (Figure 2). 0.5 µg plasmids encoding re-constructed ZFN target left and right libraries were transformed into selection strain LBS (left half binding site) and RBS (right half binding site), respectively. And 100 µl transformants were plated on non-selective plates and selective plates equally (Figure 3C). ZFN encoding plasmids were recovered from surviving colonies on selective plates to achieve efficient ZFNs specifically binding to left and right half sites. In order to express ZFNL and ZFNR in distinct plasmids, ZFPs in ZFN plasmids target right half site were transferred into pTrp-FokI between *Not*I/*Bam*HI sites for further selection.

Finally, 0.5 µg ZFNHL and ZFNHR expression plasmids were co-transformed into FBS (full binding site) selection strain bearing full target sites (Figure 3C). ZFN pair encoding plasmids were recovered and isolated from surviving colonies on selective plates. After sequencing analysis, 3 pairs of efficient ZFNs were achieved with target 24-bp sequence of goat *α s1-casein* gene.

### Activity of ZFNs Target Goat *α s1-casein* Locus

**Figure 4:**
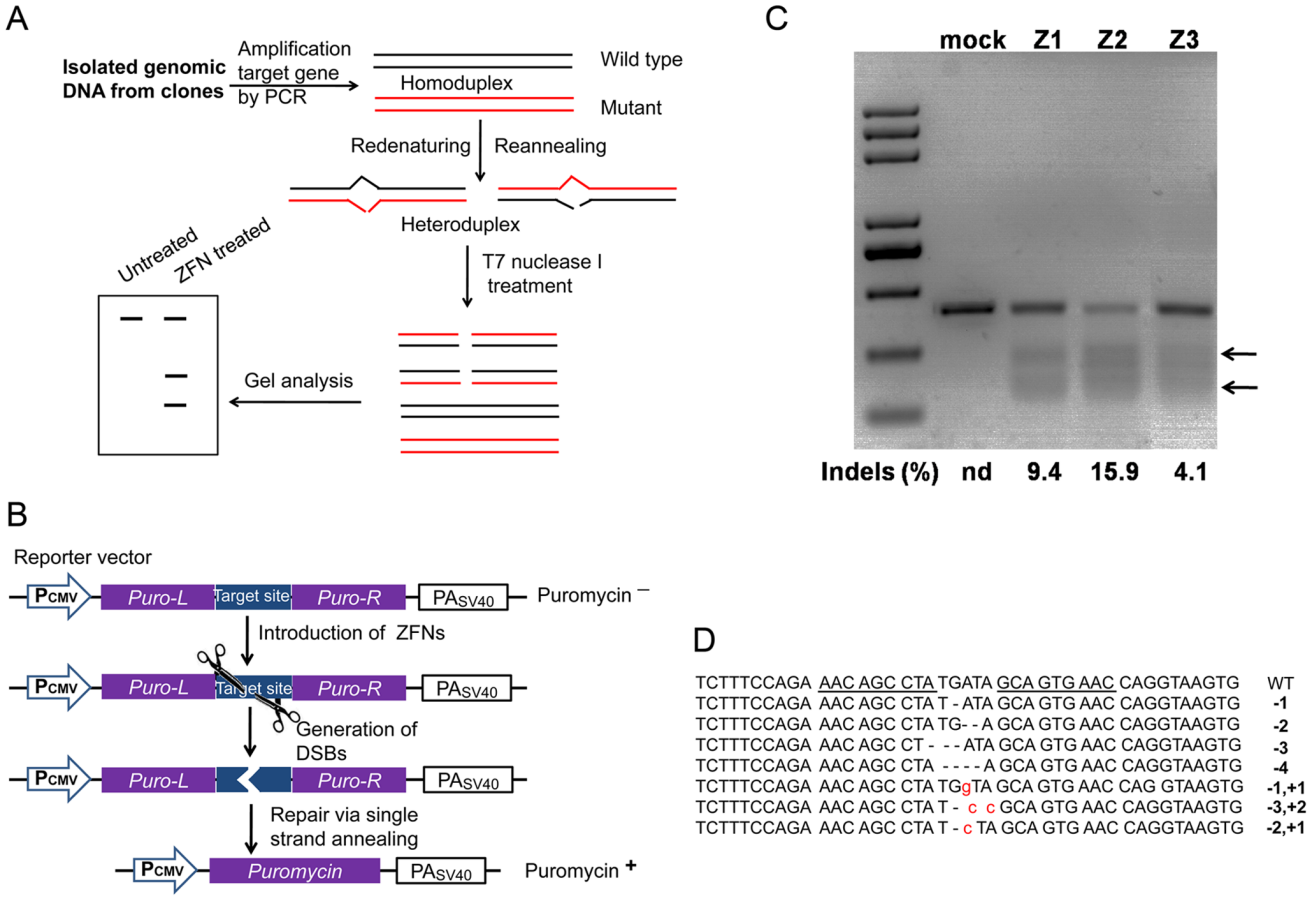
Detection of ZFNs activities targeting endogenous locus in GME cells. (**A**) **Illustration of puromycin-based system for enrichment of genome modification-positive cells.** The reporter gene of *puromycin resistance* (*PuroR*) was divided into two fragments carrying 300-bp direct repeats and a ZFN target site. The target sites in reporter vectors as well as the genome could be cut by introducing ZFNs in GME cells. Thus, restored *PuroR* conferred GME cells resistance to high concentration of puromycin pressure. Meanwhile, genomes of surviving clones were also potentially targeted by ZFNs. Pcmv: CMV promoter, Puro-L: *PuroR* left homology, Puro-R: *PuroR* right homology, PAsv40: SV40 polyA. (**B**) **Schematic representation of T7 endonuclease I assay.** Genomic DNA was isolated from surviving clones and negative control. PCR reaction was used to amplify target sequences. Then, 200 ng PCR products were re-denatured and re-annealed to generate heteroduplexes. T7 EI enzyme specifically recognizes and cleaves mismatches, and cleaved fragments were isolated by 3% agarose gel. (**C**) **Levels of endogenous locus modification mediated by 3 pairs of ZFNs.** Goat mammalian epithelial (GME) cells were electroporated with reporter and ZFNs expression vectors or control plasmids. 5 µg/ml puromycin was added to culture 2 days post electroporation, and genomic DNA was isolated 5 days after treatment with puromycin. The T7 EI assay demonstrated that 3 pairs of ZFNs Z1, Z2 and Z3 generated gene disruption frequencies at 9.4%, 15.9% and 4.1%, respectively. No gene modification was detected in GME cells electroporated with control plasmids of empty expression vectors. (**D**) **Sequences of small deletions and insertions in target site of** ***α s1-casein*** **induced by ZFNs.**

ZFNs screened by this system were further evaluated for the activity to target the endogenous gene of goat *α s1-casein* (Figure 4A). DNA double strand breaks induced by ZFNs are mainly repaired by NHEJ in the absence of donor DNA, which often introduces small insertions or deletions, designated ‘indels’, at the target site. Accordingly, PCR products of target site from genomic DNA were detected by mismatch-sensitive T7 endonuclease I assay (Figure 4B).

GME cells were electroporated with plasmids encoding ZFNs pairs and reporter plasmids. Two days after electroporation, cells were selected with puromycin at the final concentration of 5 µg/ml, which allowed for enrichment of potentially genome-modified cells, and genomic DNA was isolated. T7 EI assay demonstrated that no genomic modifications were detected in cells treated with control plasmids of empty expression vectors. Significant genomic modifications were detected in cells treated with ZFNs pairs (Figure 4C). All three pairs of ZFNs generated endogenous gene disruption at the frequencies of 9.1%, 15.9% and 4.1%, respectively. Sequencing results illustrated that indels occurred at goat *α s1-casein* locus in GME cells (Figure 4D).

## Discussion

Various selection strategies have been developed to engineer zinc fingers to bind to desired sequence with high affinity. Phage display was used to screen for Cys2His2 zinc fingers binding any 9-bp DNA sequence [34]. Cellular selection systems were also utilized to generate specific zinc finger arrays, including bacterial one hybrid and two hybrid systems [2], [20], [35], yeast one hybrid system [36], and mammalian cells [37], [38]. The problem we cannot ignore remains however that even if ZFPs recognize and bind to target sites with specificity and high affinity, ZFNs still have the risk of failure to cut the target gene *in vivo*.

Herein, we created a simultaneous screening and validation system for achieving *in vivo* functional ZFNs. This system benefits from *Gal4* reporter system and OPEN method. According to the OPEN method, we constructed three randomized 3-zinc finger nucleases, in which coding sequences of single finger were mutated with randomized primers and other two fingers were kept constant to BCR-ZFP. Based on the three ZFNs libraries, 6 report strains were constructed to enrich ZFNs target 3-bp subsites by the first step screening. Then, enriched zinc fingers were assembled together to generate two re-constructed ZFN libraries as candidate ZFNs target left and right half sites, respectively. Efficient ZFNs target left and right half sites were achieved via the second screening, and finally pairs of efficient ZFNs target 24-bp full site were obtained.

Yeast-based systems were developed for further identification of ZFN activity. One system was based on *MEL1* reporter gene to screen for ZFNs with maximal activity, which generated disrupted *ntl* alleles at frequencies averaging 20% in zebrafish [9]. Another assay was mediated by a reporter plasmid carrying *LacZ* gene, and used to validate specific ZFNs obtained through OPEN method, which induced *Arabidopsis ADH1* and *TT4* genes mutation frequencies of 7% and 16% in somatic cells [25]. Reporter genes in both assays contained two short direct repeats of coding sequence and a ZFN target site. Upon ZFNs induced DSBs at the target site, functional reporter genes were restored via SSA repair and detectable expression of restored reporter genes indicated the activity of obtained ZFNs. Both of these yeast-based systems were used to validate the ZFNs activity obtained from bacterial two-hybrid screening. Their high successful rates further support the efficiency of yeast-based system for validating ZFNs activity.

In the investigation of the false positive rate, we found that only 0.01% of co-transformants of reporter plasmids and ZFN empty expression plasmids survived on the selective plate. By contrast, about 10% of the transformants survived on selective plates in the first step screening and about 1% of the transformants survived on selective plates in the second and third step screening. These results illustrate that 0.01% false positive rate could be ignored when the true positive rates are as high as 1%. Because only one finger and 3-bp target sites were changed in the first step screening, it is easy to explain why the true positive rate of this step was much higher than positive rates in the following two steps of the screening.

In addition, we used this system to validate activities of obtained ZFNs. Two pairs of ZFNs target sheep *MSTN* gene assembled by CoDA were validated by this system, of which one pair was found to be highly efficient and the other failed to cut target sites on *Gal4* report vectors. Subsequently, active ZFNs also generated small deletions on *MSTN* in sheep skeletal muscle cells, but the other also failed to target *MSTN in vivo* (data not shown). One pair of human CCR5 ZFNs showed cleavage activity in this yeast system, and also successfully target *CCR5* gene in HEK293 cells (data not shown). These results strongly supported the concept that ZFNs activities demonstrated in this yeast system reflected their cleavage efficiency in target cells. These consistent results in yeast and mammalian cells strongly support the notion that our novel system could be applied to validate activities of ZFNs and artificial enzyme transcription activator-like effectors nucleases (TALENs) [39], [40].

To enrich genome-modified cells after ZFNs treatment, a puromycin-based surrogate system was characterized using a reporter vector containing a ZFN target site flanking two *PuroR* homology regions. Fundamentally, ZFNs cut target sites on reporter as well as genome, such that *PuroR* was restored to allow cells survival in the presence of puromycin (Figure 4A). We found that ZFN-mediated mutation efficiency was up to 15.9% with puromycin selection, although no endogenous mutation was detected without the surrogate reporter system. Obviously, the initial enrichment system could enrich cells with genomic modification.

Recently, NHEJ reporter system has been used to efficiently enrich ZFN- or TALEN-induced mutant mammalian cells [41], [42]. NHEJ-based reporter system relies on error-prone repair mechanism on double strand break sites of DNA. This surrogate reporter system is very useful for validating customer designed nucleases as one third of repaired double strand break generates positive signal by restoring shifted open reading frame. However, Karathanasis and Wilson reported that SSA via 29-bp repeat is ∼10-fold more efficient than NHEJ in *Saccharomyces cerevisiae* [43]. Wilson further demonstrated in another study that the absolute repair frequencies of NHEJ and SSA were 4.5 and 58% respectively in the wild-type strain of *Saccharomyces cerevisiae* [44]. In our system, we incorporated both SSA and NHEJ repair pathways in our reporter vector. We inserted ZFN target site into the middle of GAL4 BD and AD domains flanked by direct repeat sequence. By this design, we can pick up all of positive signals from SSA repair pathway and one third of positive signals from NHEJ repair pathway.

In conclusion, we created a system with simultaneous screening and validation of ZFNs in yeast. It is an alternative method to obtain efficient ZFNs directly and validate activities of ZFNs or TALENs readily.
